# The Generation of Phase Differences and Frequency Changes in a Network Model of Inferior Olive Subthreshold Oscillations

**DOI:** 10.1371/journal.pcbi.1002580

**Published:** 2012-07-05

**Authors:** Benjamin Torben-Nielsen, Idan Segev, Yosef Yarom

**Affiliations:** 1 Department of Neurobiology, Hebrew University, Jerusalem, Israel; 2 Edmund and Lily Safra Center for Brain Sciences, Hebrew University, Jerusalem, Israel; University of Freiburg, Germany

## Abstract

It is commonly accepted that the Inferior Olive (IO) provides a timing signal to the cerebellum. Stable subthreshold oscillations in the IO can facilitate accurate timing by phase-locking spikes to the peaks of the oscillation. Several theoretical models accounting for the synchronized subthreshold oscillations have been proposed, however, two experimental observations remain an enigma. The first is the observation of frequent alterations in the frequency of the oscillations. The second is the observation of constant phase differences between simultaneously recorded neurons. In order to account for these two observations we constructed a canonical network model based on anatomical and physiological data from the IO. The constructed network is characterized by clustering of neurons with similar conductance densities, and by electrical coupling between neurons. Neurons inside a cluster are densely connected with weak strengths, while neurons belonging to different clusters are sparsely connected with stronger connections. We found that this type of network can robustly display stable subthreshold oscillations. The overall frequency of the network changes with the strength of the inter-cluster connections, and phase differences occur between neurons of different clusters. Moreover, the phase differences provide a mechanistic explanation for the experimentally observed propagating waves of activity in the IO. We conclude that the architecture of the network of electrically coupled neurons in combination with modulation of the inter-cluster coupling strengths can account for the experimentally observed frequency changes and the phase differences.

## Author Summary

There is a profound interest in the dynamics of neuronal networks and the simulation of network models is a prevalent approach to study these dynamics. Generally, network models contain neurons that are connected mostly through chemical synapses to form either a completely regular topology (such as nearest neighbor connections), a completely random topology, small-world networks or scale-free networks. We investigate the dynamics of an atypical network, inspired by the Inferior Olive (IO) network, a brain structure located at the end of the brainstem that is responsible for timely execution of motor commands. This network is atypical in the sense that it has neurons in a clustered topology, which are connected solely by electrical synapses. The dynamics in the IO are enigmatic as the membrane voltage of some neurons can oscillate at the same frequency while maintaining phase difference with other neurons. It has also been demonstrated that propagating waves of activity occur spontaneously in this network. Using computer simulations we unraveled the mechanism underlying these previously enigmatic experimental observations. In so doing, we stress the importance of investigating more realistic network topologies to explore complex brain dynamics.

## Introduction

There is a profound interest in the dynamics of neuronal networks and the simulation of network models is a prevalent approach to study these dynamics. One aspect of network dynamics is the generation of oscillatory activity. It has been hypothesized that oscillations subserve brain-wide communications. For instance, “binding” to connect distinct sensory streams in the brain [1], [2], or entrainment of brain regions [3], [4] to facilitate communication and filtering of information [5], [6]. Computational models provide mechanistic explanations for these phenomena and explore their functional consequences. As such, electrical oscillations in the brain have been studied by using network models containing only chemical synapses [7], [8], or a mixture of chemical and electrical synapses [9]. Network oscillations (and associated experimental findings) are generally not addressed in networks connected *solely* by electrical synapses despite the fact that such brain regions, such as the Inferior Olive, exist and are known to produce oscillations. Also, most models of oscillatory neuronal activity focus on oscillatory behavior in the suprathreshold, spiking regime of neurons. In contrast, subthreshold oscillations are rarely considered outside the realm of intrinsic neuronal properties. Here we report on a network model of the subthreshold oscillations and their dynamic behavior in the Inferior Olive.

The Inferior Olive (IO) nucleus is the exclusive provider of cerebellar climbing fibers. Neurons in the IO form a network solely through electrical connections (gap junctions) between them. This electrically coupled network of neurons generates subthreshold voltage oscillations, which were observed both *in-vitro* [10]–[13] and *in-vivo* [14], [15]. Spiking activity is generally strictly phase-locked to the peaks of the oscillations. As a result of this peculiar anatomy and electrophysiological dynamics, the IO has been implicated as a timekeeper for the cerebellum and has been suggested to play an important role in the timely execution of motor commands [16]–[18] and in the generation of well-timed signals used in learning [19]–[21].

There are two observations in relation to the function of the IO as a timekeeper. The first observation is that the frequency of the subthreshold oscillation shifts from time to time [14], [22]. The base frequency of the IO subthreshold oscillation is normally well below 10 Hz and shifts of 1 to 6 Hz around the base frequency are reported [15],[22],[23]. The second observation is that while different neurons oscillate at the same frequency, phase differences among neurons are observed. Stable phase differences up to 90° between IO neurons were recorded in *in-vitro* preparations [22]. *In-vivo*, Purkinje cells complex spikes, which are considered to be the manifestation of olivary activity, displayed phase differences up to 180° [24]. The observation of phase differences in a network consisting only of neurons with direct electrical coupling is in itself problematic: how can phase differences in the subthreshold regime persist over time between two electrically coupled neurons that oscillate at the same frequency? While several theoretical models have been proposed to account for the subthreshold oscillations in the IO [10], [25]–[29], none of these works provided an explanation for the controllable modulation of frequencies or for the generation of persistent phase differences.

In this work we address both frequency modulation and the generation of phase differences in the IO network. To this end we built a network model of the IO consisting of basic conductance-based model neurons [30] in an architecture based on anatomical and physiological data. The model neurons contain leak (g_l_) and low-threshold Ca^2+^-conductances (g_Ca_, see Methods). At particular densities of these two conductances, the neuron model exhibits spontaneous oscillations [30]. Anatomically, it is known that somata of IO neurons cluster together in small groups of 8–12 neurons [10], [31]. This causes considerable overlap between the dendrites of neurons from the same cluster. In turn, this overlap gives rise to many dendro-dendritic gap junctions between neurons of the same cluster. Because of the limited space in which neurons are situated, there is, arguably, less overlap between dendrites of neurons belonging to different clusters. Hence, gap junctions are less frequent between neurons of different clusters. Additional details about the connectivity come from physiological experiments in which pairs of IO neurons are recorded simultaneously. It is known that each neuron connects to 1–38 other neurons [1], [2] and that the coupling coefficient (CC_1_ = V_2_/V_1_, CC_2_ = V_2_/V_1_, and see Methods) ranges from 2–20%. Although nearby neurons are more likely to be connected, the strength of individual connections is only weakly correlated with distance from the soma. There is also physiological support for nearby neurons having similar biophysical features, such as the density of low-threshold calcium conductances. The experimental support is indirect and stems from two different lines of evidence. First, *in vitro* preparations show that nearby neurons oscillate with the same phase and frequency [32]. Since the coupling strength between neurons is notoriously low, such similar oscillations can only occur when the neurons share the same conductance densities that drive the oscillation. Second, the coupling coefficient between nearby neurons is symmetrical [33] – a feature that only results from neurons with equal input resistances. As the input resistance at rest is mainly determined by the leak and low-threshold calcium conductances (in combination with the h-type conductance), the densities of these conductances must be very similar.

These data constrain the model's architecture to a topology in which similar neurons (in terms of conductance densities) are clustered together and are densely connected via gap junctions. The anatomical clustering of dendrites leads to sparse connectivity between a given cluster and all other clusters, i.e., neurons from one cluster are connected to neurons in one or a few other clusters but not necessarily to all other clusters. Thus, major constraints on the network architecture are imposed by the connectivity scheme, the limited number of connections per neuron, and the weak coupling coefficient between cell pairs.

We demonstrate that network models which obey these experimental constraints, and in which electrical-coupling strength is subject to modulation, are sufficient to account for frequency changes and for the generation of phase differences across frequencies. The robustness of the results is discussed and the key mechanisms that support the observed network dynamics are highlighted. We also discuss a prediction based on our theoretical study.

## Results

### Constructing a network model based on experimental data

**Figure 1:**
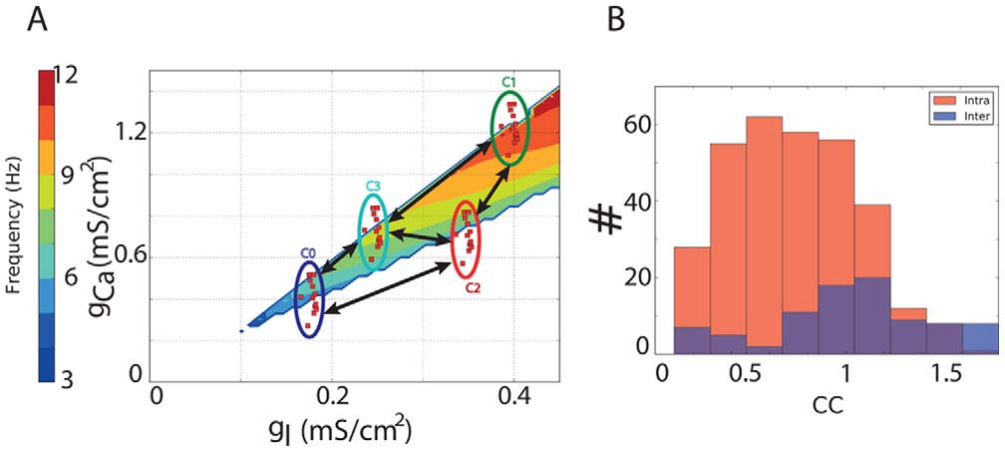
Proposed network architecture. A: Model neurons only contain leak and Ca^2+^ currents and spontaneously oscillate at frequencies determined by the exact density of the associated conductances. Colors of the g_l_-g_Ca_ plane indicate the frequency at which a model with the corresponding density of conductances oscillates; in the white region model neurons do not oscillate spontaneously. The network itself consists of individual neurons (red squares) grouped in clusters (colored ellipses; color not related to the frequency). Neurons inside the cluster are connected to 4 neighbors. When two clusters are connected (black arrows) each neuron from one cluster is connected to a random neuron in the other cluster. All connections are gap-junctions. B: Resulting coupling coefficients of all connections in the network. This specific network is used throughout the manuscript for demonstration purposes.

The aforementioned constraints still leave several free parameters. The exact number of neurons in a cluster is bounded by biological data (8 to 12 neurons per cluster [31]), but not uniquely defined. Also, the number of clusters is variable and might be dynamic as there is evidence for dynamic control of the effective coupling strengths between clusters [15]. Since there is a hard limit on the maximal number of connections per neuron (38, from [2]), the actual number of connections per neuron varies with the cluster size and the number of clusters. In this work we devised a reference network of 4 clusters, each containing 12 neurons. The structure of this network within the g_l_-g_Ca_ space is shown in Figure 1A. Only the oscillating area is marked and the frequency of the oscillations is color-coded (for further details see Supporting Text S1). Cells are marked as red squares and clusters are delineate by ellipses. We limited ourselves to four clusters for the sake of clarity. To satisfy the connectivity constraints, we connected each neuron inside a cluster with 4 peers. To simulate a connection between two clusters, we connected 80% of the neurons in one cluster with a matching number of randomly selected neurons in the other cluster. The conductance of the gap junctions was chosen so as to result in a coupling coefficient of 2–20% (Figure 1B). In Figure 1B the coupling coefficient of each intra-cluster connection (red) and each inter-cluster connection (blue) in the network is illustrated. Note that we provide two CCs per connection because the inter-cluster CC is asymmetrical due to differences in the input resistances of connected neurons. Clustering is organized in such a way that neurons inside each cluster share similar conductance densities. For the sake of demonstration, we picked the clusters in such a way that they were on the boundary in parameter space where neurons can either display spontaneous oscillations or not. We picked neurons on this boundary because the robustness of the oscillatory behavior suggests that at least some of the neurons behave as spontaneous oscillators. On the other hand, stable, non-oscillating, neurons are also encountered [1], [23].

### A data-driven clustered network generates stable subthreshold oscillations

**Figure 2:**
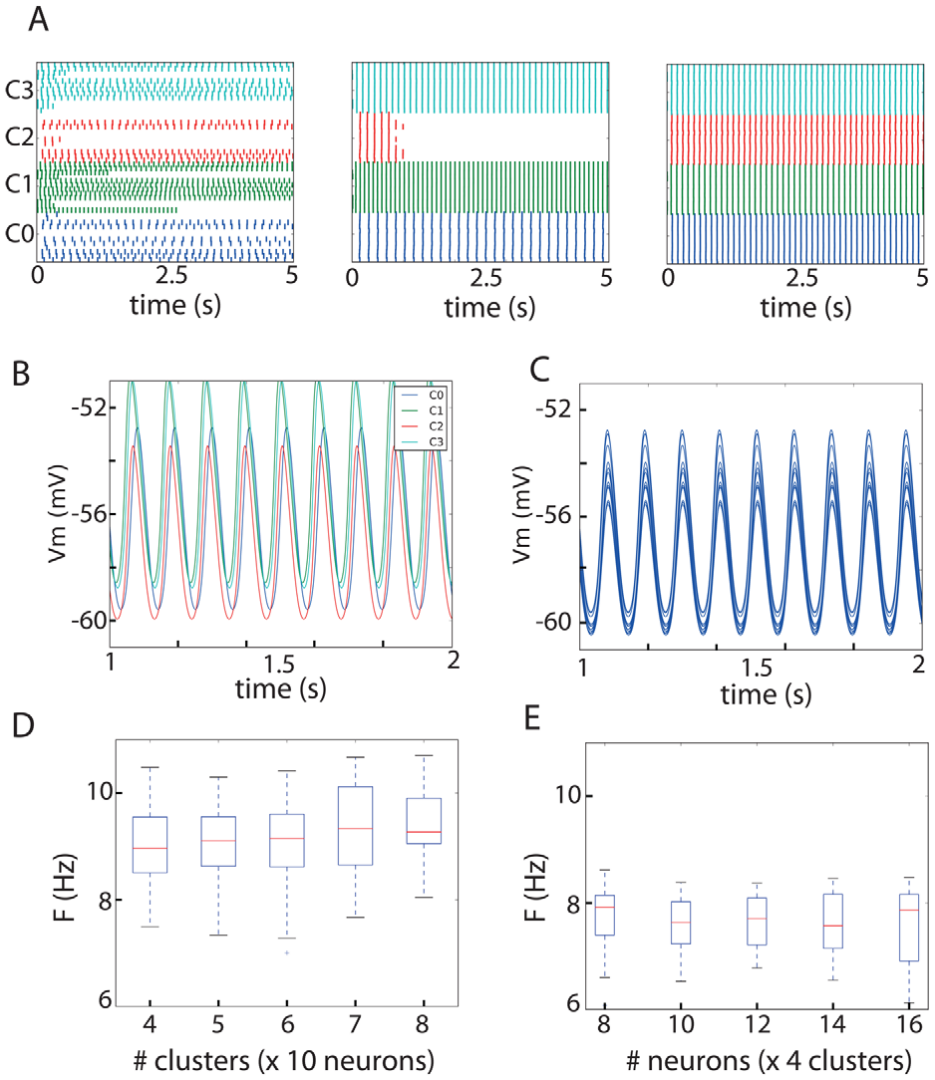
Stable subthreshold oscillation in a clustered network of the IO. A: Raster plot containing all neurons in the network; peaks of the oscillation are denoted by a dot. Without connections only 26 out of 48 neurons oscillate (left panel). When the intra-cluster connections are added, 3 out of 4 clusters show synchronized oscillations within the clusters (center panel). After adding the inter-cluster connections as well, the whole network reaches a synchronized oscillation of 9.2 Hz. B: Detail of the membrane potential of one neuron from each cluster indicating that the network can sustain stable subthreshold oscillations. Colors of the membrane trace and the ellipses in panel A are matching. C: Detail of the membrane potential of all neurons in one cluster (C0). D&E: Stable oscillations in the proposed network architecture are robust to changes in the number of clusters and the number of cluster per neuron. In D, networks with a varying number of clusters but a fixed cluster size (10 neurons) and a randomized connectivity scheme were tested. In E, networks with 4 clusters and a varying cluster size were tested (while the connectivity scheme was fixed as in the reference network. Therefore, the “4 clusters×10 neurons” from panel D and E are not the same). Boxplots indicate the median and the boxes extend from the lower to the upper quartile. It follows that robust synchronized oscillations can be generated by a variety of networks and that each network can achieve a range of frequencies.

In our reference network, the conductance densities of twenty-six out of forty-eight model neurons are such that they oscillate spontaneously (Figure 2A, left panel). After adding intra-cluster gap junctions in accordance with the connectivity scheme described above, all neurons in clusters C0, C1 and C3 started oscillating, whereas the oscillations in cluster C2 diminished within 1 second (Figure 2A, center panel). With further addition of the inter-cluster gap junctions, all neurons in the network started oscillating and the network exhibited stable oscillations (defined as non-dampening over 5 s) at a frequency of 9.2 Hz (Figure 2A right panel and Figure 2B). Close examination of these oscillations revealed that neurons within a cluster oscillate at precisely the same frequency and phase (Figure 2C), whereas phase differences were evident when neurons from different clusters were compared (Figure 2B). The amplitude of the subthreshold oscillations is less constrained in the experiments and varies on a cell-to-cell basis. However, as indicated by its name, the peak of the oscillations should remain in the subthreshold regime and not provoke suprathreshold events. The simulated voltages observed in our simulations fit nicely with the experimentally observed range of 0.5–25 mV [1], [12], [22]. We use the term “synchronized oscillations” to describe the network state in which all neurons oscillate at the same frequency (but not necessarily with the same phase).

It is important to stress that the network dynamics are robust with respect to the free network parameters (i.e., the exact number of clusters and the cluster size), as long as the resulting connectivity pattern meets the anatomical and physiological constraints outlined before. Namely, we can obtain different networks composed of various numbers of clusters and cluster sizes that exhibit synchronized oscillations. To support this claim, we simulated two sets of pseudo-random network. In the first set, we simulated networks consisting of 10 neurons per cluster and varied the number of clusters from 4 to 8. The inter-cluster connectivity scheme was also sampled randomly, with each cluster connecting to 1–3 other clusters. In the second set of simulations, we varied the number of neurons inside each cluster between 8 and 16, while keeping the number of clusters constant, and using a fixed inter-cluster connectivity scheme as in the reference “4 clusters×12 neurons” network. The resulting frequencies at which these networks exhibited spontaneous oscillations are shown in Figures 2 D & E, respectively. In both sets of simulations, the actual conductance densities of each neuron were sampled from within the experimentally observed range, and the actual gap junction conductances were sampled so as not to violate the strict constraints on coupling coefficients between neurons. We found that the generated networks displayed stable, synchronized oscillations in a wide variety of frequencies. Note the difference in the results between the two “4 clusters×10 neurons” simulations shown in Figures 2 D & E. This difference stems from the distinct inter-cluster connectivity schemes.

We also want to stress that roughly 50% of neurons in our “4 cluster×12 neurons” reference network oscillate spontaneously. Evidently, the mechanism we presented for generating synchronized oscillations also holds in networks with a higher proportion of spontaneously oscillating neurons (e.g., 85%, as in [15]). We thus show that our network model is able to mimic the experimentally observed subthreshold oscillations, and that the “4 clusters×12 neurons” reference network is a good representative of a larger set of networks that satisfies the experimental constraints. (Also see Supporting Text S1)

### Inter-cluster coupling strength modulates the frequency of network oscillations

**Figure 3:**
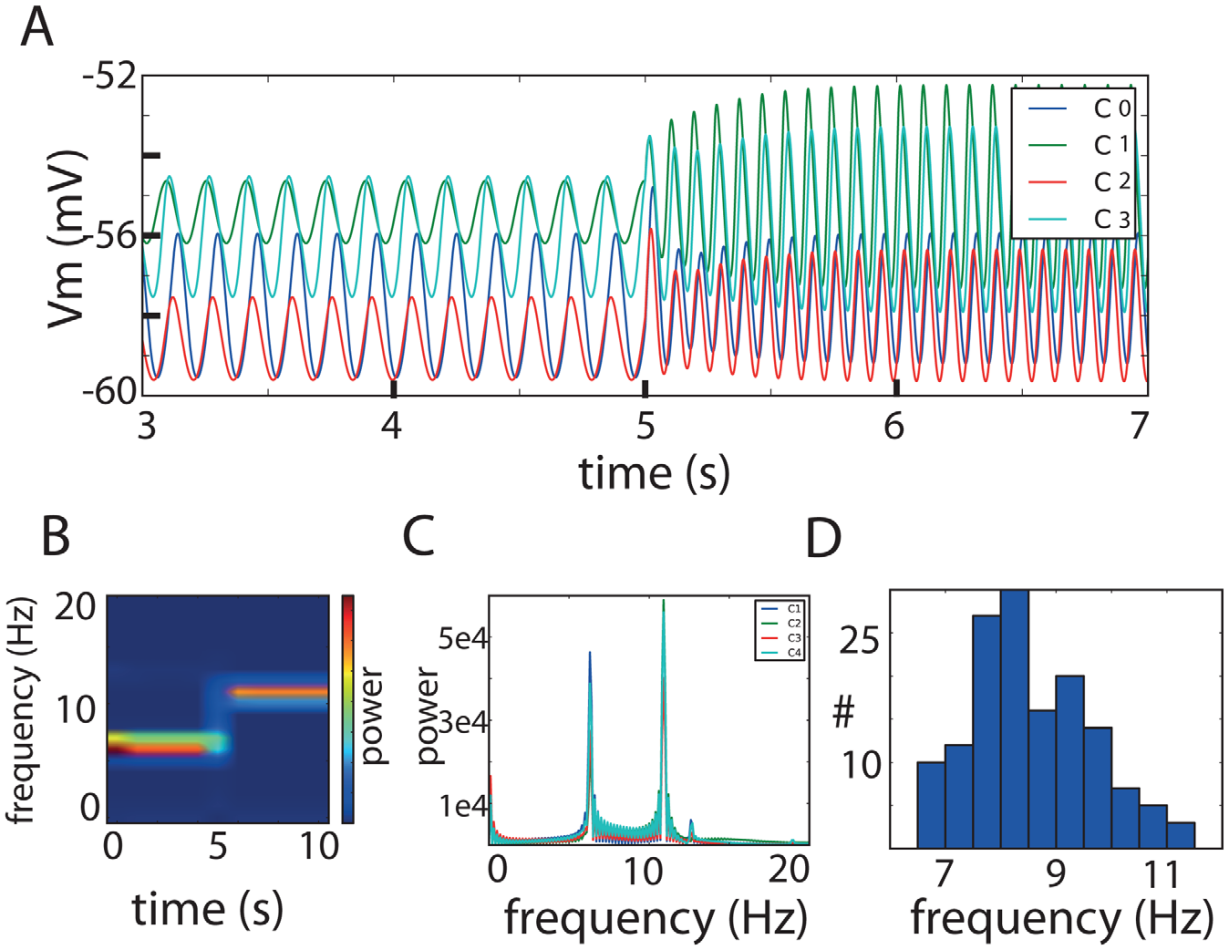
Robust modulation of network frequency by changing the inter-cluster coupling strengths. A: Membrane potential of one neuron per cluster just before and after manually changing the inter-cluster connection strength in the reference network. The change in inter-cluster strength caused a shift in the synchronized oscillation frequency from 6.3 Hz to 10.9 Hz. B: Short-term Fourier transformation of the membrane potential of one neuron in the network indicates the shift in frequency. C: Fourier transformation of the membrane potential of one neuron of each cluster. All clusters oscillate at the same frequency and are subject to the same shift. D: Histogram of frequencies at which the same network with pseudo-random inter-cluster connections strengths can oscillate in synchrony. Only changing the inter-cluster coupling strength (within realistic ranges, i.e., CC<20%) can be sufficient to bring the network to a state of synchronized oscillations with frequencies between 6 and 11 Hz.

Two model IO neurons are known to be able to oscillate synchronously when they are connected with a suitable coupling strength [30]. Moreover, it was previously found that such a pair would behave as a single neuron that contains the average density of the conductances of both neurons. The same mechanism also works for networks of IO model neurons. Indeed, we show that the reference network can exhibit oscillations between 6–12 Hz upon modification of the electrical coupling strength. Figure 3A shows the voltage in four neurons: one from each cluster. At the beginning of the simulation (t<5 s) the network oscillates at 6.3 Hz, and after modulating the connection strength (at t = 5 s), the network oscillates at 10.9 Hz. We changed the coupling strength in a biologically plausible way. Although the exact conductance change of each connection was randomized, the changes of all the connections between two clusters followed the same trend and either decreased or increased. This way, heterogeneity was maintained. The changes were always limited to a sevenfold decrease/increase of the present conductance. In the reference network, the modulation consisted of strengthening the connections from groups C3 and C4 to group C1 up to sevenfold, while moderately decreasing their connection strength with C0 by a factor of up to four. Intuitively, the frequency at which the network synchronously oscillates is the frequency of the “center of mass” of the connected neurons, i.e., the frequency of the weighted average (in terms of the conductances) of all connected neurons in the network. The reported shift in network frequency can then be interpreted as a shift of the “average neuron” on the g_l_-g_Ca_ plane (Figure 1A) from bottom left to top right. The frequency change can be verified by a short-time Fourier transformation (Figure 3B) and the standard Fourier transformation (Figure 3C). As a second step we assessed the robustness of the mechanism that modulates the network frequency by repeatedly changing the inter-cluster strength. For this purpose we simulated a large number of instances of the same “4 cluster×12 neurons” network but with different inter-cluster connection strengths. Additionally, we also changed the coupling coefficients randomly by 20% to 400% during simulation of the network (while still staying within the limit of CC<20%). By doing so we found networks displaying synchronized oscillations in the 6–11.5 Hz frequency range both before and after changing the connection strengths (Figure 3D).

Thus, we identified a robust mechanism to change the frequency of the synchronized oscillations by means of (small) changes of the inter-cluster strengths that in turn change the weighted-average neuron that dictates the frequency of the synchronized oscillation.

### Phase difference between clusters during stable oscillations

**Figure 4:**
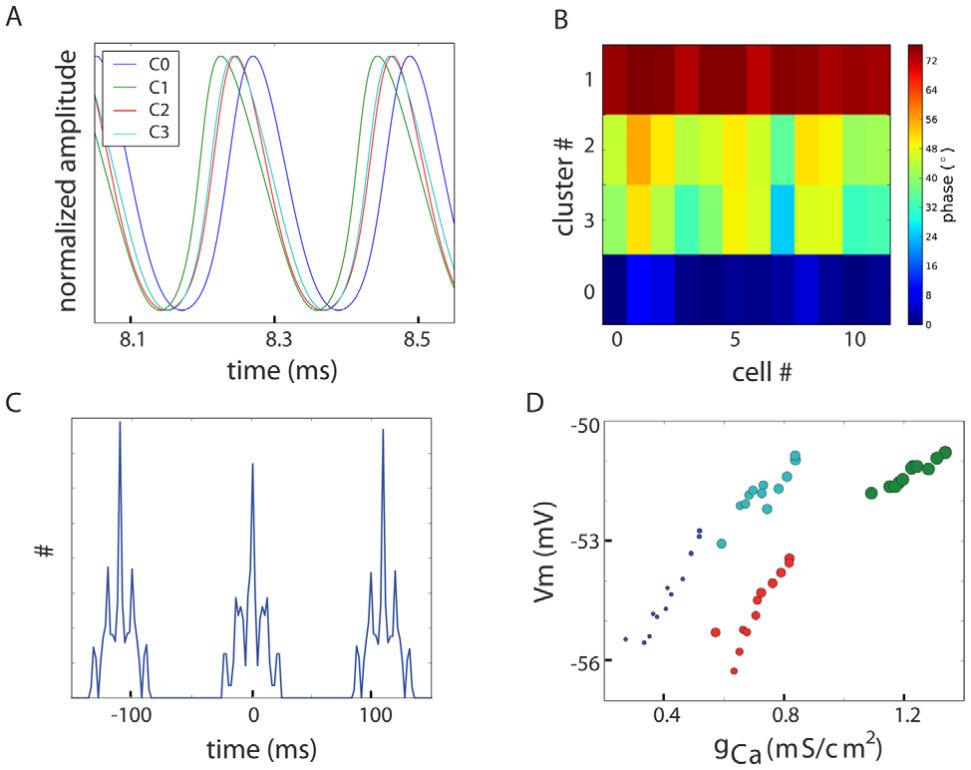
Stable phase differences between neurons. A: Focus on the normalized membrane potential of one neuron per cluster reveals that clusters with higher Ca^2+^-conductance are advanced in phase with respect to other clusters (traces have colors matching with Figure 1). In the regime of oscillatory IO neurons, higher Ca^2+^-density indicates a higher resting membrane potential that causes the neuron to lead in the phase. B: Phase-map color coding the phase-difference between all neurons in the network. Phase differences are given in degrees relative to the inter-peak-interval; the phase of the bottom left neuron is taken as reference (0°). Neurons within the same cluster have similar phases due to similar resting potentials, while larger phase-differences arise between clusters that are farther apart in terms of their conductances. The maximum phase-difference between two neurons was 72° in the demonstration network. C: Cross-correlation of the peak times between (one neuron from the) four clusters computed for 5 s traces confirms that the phase-differences are stable over time. D: The amplitude and phase difference is proportional to the amount of g_Ca_-conductance a neuron contains. The y-axis denotes the peak voltage and the x-axis indicates the conductance density. The color-coding is the same as in A while the size represents the phase-difference (as measured between the neuron at the bottom left and any other neuron).

An emergent feature of the proposed clustered network architecture is that such networks display a phase difference between neurons (Figure 4A). This phase difference is a consequence of the difference in the ion channel density in each cluster. The voltage build-up in neurons with a higher density of Ca^2+^-conductance is faster. As a result, these high Ca^2+^-conductance neurons oscillate at a higher frequency when uncoupled. In the coupled case, the faster voltage build-up leads to their advance in phase over neurons with less Ca^2+^-conductance. During the period directly after the peak, the current flowing between both neurons reverses and causes both neurons to remain in pace with each other. When the coupling strength is sufficient, it is this mechanism that binds the two connected neurons to the same frequency. The same principle holds for networks with clusters of similar neurons: the cluster with highest concentration of Ca^2+^-conductance is advanced in phase over clusters with less Ca^2+^-conductance. Figure 4A shows the membrane potential of a representative neuron for each cluster, illustrating that while the network oscillates in synchrony, the temporal succession of the voltage peaks corresponds to the decrease in Ca^2+^-density (the colors of the traces match the colors of the clusters in Figure 1A.) The observed phase differences in the reference network are summarized in Figure 4B. The respective phase of each neuron is color-coded with respect to that of the reference neuron. It can be verified that within a cluster, the neurons oscillate at roughly the same phase, whereas a larger phase difference exists between different clusters. In the 9.2 Hz regime, the maximum phase difference between any pair of neurons was 72° (Figure 4B). The aforementioned phase difference is stable inasmuch as the phase relations between neurons are maintained over a period of time. This stability over time is illustrated by the cross-correlation between the peak-times (as done with spike times) of the different clusters (measured between one neuron from each cluster and over the 4 seconds of simulated time, Figure 4C). We assessed the robustness of this phenomenon by analyzing the data from the previously generated variants of the reference network (from Figure 3D) and found that the maximal phase difference observed was 140°. Most inter-cluster phase differences were between 20° and 130° (data not shown).

The implication that neurons advanced in their phase also have higher voltage amplitude (because of the larger g_Ca_) can be verified using Figure 4D. In this figure, the peak voltage of all neurons is plotted against their g_Ca_-density. The size of the data points indicates the phase difference relative to the reference (0° phase difference). Hence, larger data-points in Figure 4D indicate a greater offset of phase with respect to the reference neuron. The number of gap junctions and the connectivity between neurons also play a role in the generation of phase differences: the gap junction in itself changes the input resistance (which in our model neurons is a manifestation of the leak conductance). This different connectivity results in a different number of gap junctions, which can account for the difference between clusters 2 and 3 in Figure 4C.

The observed phase difference also provides an explanation for the “propagating waves of activity” found experimentally [22]. In the event that there is spatial correlation between the clusters, different clusters will be activated sequentially, in descending order of g_Ca_. This sequential activation can be observed as a propagating wave (see Supporting Text S1 and Supporting Video S1).

Thus, our model also successfully reproduces the experimental observation of phase differences, and provides a mechanistic explanation for this phenomenon.

## Discussion

In this work we proposed a plausible model of the IO network that provides an explanation for timing and timekeeping within the IO. The activity in the IO is crucial for the proper function of the olivo-cerebellar circuit, and as such it is at the focus of many studies. Different models of IO neurons have been proposed to explain single-cell subthreshold oscillations [30], complex firing dynamics [29], the influence of dendritic spines on synchrony [25] and rhythmogenesis [26], [28]. The dynamic formation of clusters and transient phase differences were demonstrated to emerge from chaotic dynamics [34]. To our knowledge, our IO network model is the first model to reproduce previously unexplained experimental findings such as the non-chaotic, controllable frequency changes and the generation of phase differences, and to provide a mechanistic explanation for these findings.

We purposely used minimalistic model neurons, as the focus of this work was the dynamics of the subthreshold oscillations in the IO network. The model neuron contains only a leak and a Ca^2+^-current because these currents are most prominent in the subthreshold voltage oscillation regime ([−65 mV,−50 mV]) [29], [30]. Clearly, there are many other voltage-gated ion-channels expressed in IO cells that were not included in this study [29], [35]. However, these channels mostly affect action potentials (especially, the characteristic high-threshold Ca^2+^ spikes). These currents could be added in the future in large-scale models of the olivo-cerebellar circuit. Despite its limitations, our model is elegant in its minimalistic, yet biologically rooted approach.

In this work we re-evaluate a finding from an earlier work in which it was shown that two IO model neurons that are not necessarily oscillatory in isolation can be connected in such a way that they oscillate synchronously [30], and we interpret this result in a network context. Previously, it was shown that, in the limit of strong coupling, a pair of IO model neurons could be considered as a single neuron containing the average conductance of both individual neurons. Consequently, the frequency of the synchronous oscillation in a pair of such neurons is determined by the frequency of the hypothetical average neuron [30]. Manor et al. proposed as a rule of thumb that an electrically coupled pair of IO model neurons will oscillate only when the “average neuron” lies in the region of the g_l_-g_Ca_ plane where a single neuron would oscillate spontaneously [30] (i.e., inside the colored region in Figure 1A). We continued to show that the same mechanism holds for a network of IO model neurons. In that sense, and as we demonstrated, the inter-cluster connection strength dictates the frequency of the synchronized oscillations because it weighs the contributions of each cluster to the average neuron. In the Supporting Text S1 we provide analytical and empirical support for the demonstrated effects of coupling strength on the frequency of synchronized oscillations.

Having shown that the inter-cluster coupling strength determines the frequency of oscillation, it is straightforward to see that changes in the inter-cluster coupling strength change the oscillatory frequency in the network. We note that the intra-cluster coupling strength does not contribute to the network frequency because inside a cluster all neurons are electrically similar and hence the average neuron that represents a single cluster is very stable; only the inter-cluster connections can change the frequency. We also note that in the clustered network as we propose it, the synchronized oscillations can cease in two ways. First, the virtual, weighted average (neuron) can be moved to a region in the g_l_-g_Ca_ space were no oscillations occur (i.e., the white space in Figure 1A). In this case the whole network is stable and no oscillations occur in any of the neurons. Second, the coupling coefficient between particular clusters can be decreased to a point that their mutual influence is too low to sustain synchronized oscillations. In this case the network breaks down into smaller functional units in which oscillations may persist, albeit with different frequencies. The second mechanism allows for resizing and reassembling the functional network in which synchronized oscillations occur.

Changes in the functional coupling strength can be induced by the GABAergic inputs coming from the deep cerebellar nuclei (DCN). DCN inputs to the IO are co-located at the sites of the gap junction [17], [36] and can shunt the current between two neurons [32], [37]–[39]. Increased input from the DCN can thus serve to decrease the coupling strength, while a release from (tonic) inhibition can increase the coupling strength [39], [40]. Thus, a whole range of coupling strengths can be achieved between clusters, which can result in a continuum of frequencies at which the network can oscillate in synchrony. Our proposed mechanism contrasts with the mechanisms proposed in [15], in which discrete network frequencies result from coupling and decoupling of individual neurons.

Blocking of GABAergic inputs has been reported to have the effect of increasing the size of the group of synchronously oscillating neurons [12], [32], [41]. Thus, apart from the effect of modulating the frequency, GABA could also modulate the size of the group of synchronously oscillating neurons, which in turn has an effect on the coherence in Purkinje cell activity. Our model also captures the re-arrangement of the group of synchronously oscillation neurons. In Figure 2A (center panel), the network activity is shown when only intra-cluster connections are present, which effectively mimics a situation in which clusters are uncoupled by GABA. Then, when we add the inter-cluster connections (effectively mimicking blocking of GABAergic inputs), the complete network goes into a state of synchronized oscillations (Figure 2, right panel). Thus, our network model also captures the effect of blocking GABA, which increases the number of coherently oscillating neurons.

We found that basic neuron models including one active component (Ca^2+^ T-type current) in combination with a clustered network with differential inter-cluster electrical connections can account for synchronized network oscillations, the modulation of the frequency and the emergence of phase differences, which in turn lead to propagating waves of activity. There is a great deal of theoretical literature related to synchrony in neural network [42]–[45]. Synchrony of suprathreshold dynamics (spikes) is often explained in terms of the coupling functions between neurons [43]–[46]. On the other hand, synchrony between the subthreshold dynamics in neurons has received less attention and is rarely considered in isolation from its suprathreshold counterpart, despite the fact that this is exactly what happens in the IO, in which the firing rate is an order of magnitude slower than the subthreshold oscillations. Theoretical studies are well suited to find transitions in dynamics (bifurcations) and allow researchers to pinpoint the necessary conditions for particular experimental observations [47]. To our knowledge, there is no study illustrating the conditions required for a network to maintain non-zero phase lags between purely subthreshold oscillations. We presented a network in which such non-zero phase lags are exhibited and explained their existence in terms of the biophysics of voltage-gated Ca^2+^ current. However, it remains unclear what the minimal conditions are for realistic, synchronized subthreshold oscillations in our network. The minimal conditions depend on what is functionally relevant for the network. For instance, shifts between 1 and 4 Hz have been observed experimentally [22]. Clearly, as demonstrated in our network model, the difference between the intrinsic frequencies of any cluster in the network will place an upper bound on the size of the shift achievable in that network. As a rule of thumb, the maximum shift in a network is limited by the difference between the intrinsic frequencies (uncoupled) of the clusters (Figure S3 in Text S1). Thus, to create a shift of 2 Hz in the network, the intrinsic frequencies of the contributing clusters should be at least 2 Hz apart. However, there is a trade-off between the magnitude of the shift and the ability of the network to synchronize: the more dissimilar the intrinsic frequencies of the clusters, the harder it becomes to create coherent oscillations across the entire network (Figure S2B in Text S1). A second rule of thumb is that to synchronize two highly dissimilar neurons or clusters (say, F1–F2>2 Hz), synchrony can be obtained more easily by introducing an intermediate neuron or cluster. Consequently, the minimal conditions for a network to synchronize depend on the exact requirements, e.g. the frequency of the synchronized oscillations and the size of the frequency shift. For now we offer the aforementioned rules of thumb, but finding the precise minimal conditions required for synchrony will be addressed in future work.

Many network models are devised to address a particular question dealing with a part of the natural, experimentally observed dynamics. To model different dynamics in the same system, a new model is constructed in the present study that can accommodate diverse sets of dynamics. We have shown that our network model, which successfully reproduces subthreshold oscillations, also accounts for the experimentally observed frequency changes and phase differences. Moreover, based on current data from the DCN [40], it is a plausible that the actual connectivity between the DCN and the IO could implement the proposed mechanism of IO frequency modulation. No structural changes (such as a different connectivity statistics) are required in our model in order to generate oscillations, to change the frequency and to maintain stable phase differences between different IO cells. The fact that our model can reproduce a variety of experimentally observed behaviors increases our confidence that we have captured in our model the key mechanisms underlying the observed behavior.

The results presented in this study also give rise to a testable prediction about the IO. Our prediction addresses the possibility of modulating IO oscillation frequencies by changing the inter-cluster coupling strength. This prediction could be tested in an *in-vitro* preparation in which a single intracellular recording is made from an IO neuron while GABAergic input is emulated by GABA application. We predict that when GABA is released in small areas close to the dendrites of the recorded cell, a reversible change in the frequency should be detected. The aim would be to apply GABAergic input only to the dendrites to shunt some of the gap-junctional current while maintaining the rest, thus leaving the intrinsic dynamics of the cell largely unaffected. Consequently, the neuron would not be uncoupled completely from the network, but the influence from the network would change. This corresponds to changing the inter-cluster coupling strength and should affect the oscillatory behavior of that neuron.

In conclusion, we present the first anatomically and physiologically plausible (albeit reduced) network model of the IO that provides a biophysical explanation for previously unexplained experimental observations. As such, we believe that our model is suitable to test future hypotheses about the origin of the subthreshold oscillations and their role in timing.

## Methods

### Model neuron

We use conductance-based model neurons based on the model presented in [30]. These conductance-based model neurons contain only a leak current and a low-threshold (T-type) Ca^2+^ current. Formally, the dynamics of the model neurons are described by:
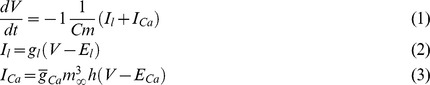
in which Cm is the membrane capacitance, E_l_ and E_Ca_ are the reversal potentials for the leak and low-threshold Ca^2+^ current, respectively. g_l_ and g_Ca_ are the maximum conductances of these currents. m and h are the gating variables for the time and voltage dependent T-type current and follow
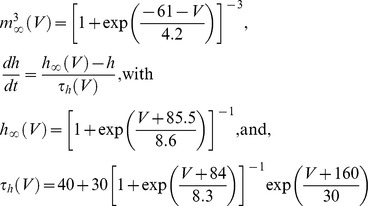



In all presented simulations, E_L_ = −63 mV while g_l_ and g_Ca_ vary between [0.15,0.4] mS/cm^2^ [0.2,1.4] mS/cm^2^ [23], respectively. Neurons containing specific amounts of g_l_ and g_Ca_ can exhibit spontaneous oscillations over a range of frequencies as illustrated in Figure S2 in Text S1. A model neuron can be equipped with different densities of the associated leak (g_l_) and calcium (g_Ca_) conductance. Depending on the exact density of g_l_ and g_Ca_ the neuron can be i) a spontaneous oscillator and oscillate at different frequencies (Figure 1), ii) a conditional oscillator, iii) bistable or, iv) stable [30].

### Network model

We create the network model by connecting selected neurons through electrical coupling (gap-junctions). The effect of a gap-junction on a single neuron can be represented by an additional current that mimics the current flowing between two connected cells proportionally to the difference in membrane potential in both cells: 
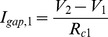
 and 
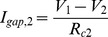
, which is added to the right-hand side of the appropriate equation (1). The precise values of R_c1_ and R_c2_ are of little importance as they depend on the actual input resistance of a neuron. A more useful measurement of coupling through gap-junction is the coupling coefficient: CC_1_ = V_2_/V_1_ = R_2_/(R_2_+R_c1_) and CC_1_ = V_2_/V_1_ = R_1_/(R_1_+R_c2_) as it directly assesses the electrical impact of one neuron on the other. Note that the voltages V_1_ and V_2_ are not the same in the calculation of CC_1_ and CC_2_ because they are measured from two separated experiments; one in which the current is injected in the first neurons and another experiment in which the current is injected in the second neuron. Due to the dependence on the input resistances, CC_1_ and CC_2_ also do not need to be the same.

Based on anatomical and physiological data the network architecture has to satisfy three interconnected constraints. First, neurons similar in terms of their conductances densities are clustered together and connected more densely to neurons inside the same cluster than to neurons belonging to different clusters. Second, the number of connections per neurons is between 1 and 38 [2]. Third, the connection strength is limited to a coupling coefficient between 2 and 20%. However, the majority of connections have a reported strength of CC<10% [1].

We generated pseudo-random networks in which we manually set the meta-parameters of the network, namely the number of neurons per cluster (12), the number of clusters (4), the number of connected neighbors inside a cluster (4), the overall connectivity scheme between clusters (Figure 1B), and, the number of connections between 2 connecting clusters (1 per neuron). In the networks generated for Figure 2 D&E, we sampled one cluster center for each cluster. We then sampled according to a normal distribution around this center (μ = 0.005 mS/cm2 and μ = 0.01 mS/cm2 for g_l_ and g_Ca_, respectively) to get set the actual values for the conductances of the model neurons inside that cluster. The networks in Figure 2D have a randomized connectivity scheme in which each cluster was connected to one to three other clusters. The networks in Figure 2E had a fixed connectivity scheme, namely the scheme from Figure 1 (left). The networks in Figure 3D were the same as the reference network and only differed in their inter-cluster strengths.

We implemented all simulations in PyNEURON [48]; the code is available on ModelDB (accession number: 144502). Analysis of the network dynamics was done with custom routines in Python/SciPy/Matplotlib (Python: http://python.org, SciPy: http://www.scipy.org/, Matplotlib: http://matplotlib.sourceforge.net/). The “phase-map” in Figure 4B is generated by computing the phase difference between each pair and setting the first neuron in the first cluster as the reference (i.e., 0° phase-difference). For the visualization, the clusters were ordered from bottom-to-top in order of larger phase-difference to the reference. The cross-correlation in Figure 3C is computed from the peak times (as is generally done with spike times) and not from the full membrane potential trace.

## Supporting Information

Text S1 Additional information about the robustness of the model, modulation of network frequencies and the range of frequencies at which (a pair of) IO model neurons can oscillate.
(DOC)

Video S1 Video illustrating propagating waves of activity in a “20 clusters×20 neurons” network.
(MPG)

## References

[1] Devor A, Yarom Y (2002). Electrotonic Coupling in the Inferior Olivary Nucleus Revealed by Simultaneous Double Patch Recordings.. J Neurophysiol.

[2] Hoge GJ, Davidson KGV, Yasumura T, Castillo PE, Rash JE (2011). The extent and strength of electrical coupling between inferior olivary neurons is heterogeneous.. J Neurophysiol.

[3] Brockmann MD, Pöschel B, Cichon N, Hanganu-Opatz IL (2011). Coupled Oscillations Mediate Directed Interactions between Prefrontal Cortex and Hippocampus of the Neonatal Rat.. Neuron.

[4] Roš H, Sachdev RNS, Yu Y, Šestan N, McCormick DA (2009). Neocortical Networks Entrain Neuronal Circuits in Cerebellar Cortex.. J Neurosci.

[5] Solinas S, Nieus T, D'Angelo E (2010). A realistic large-scale model of the cerebellum granular layer predicts circuit spatio-temporal filtering properties.. Front Cell Neurosci.

[6] Marshall SP, Lang EJ (2004). Inferior Olive Oscillations Gate Transmission of Motor Cortical Activity to the Cerebellum.. J Neurosci.

[7] Bartos M, Vida I, Frotscher M, Geiger JRP, Jonas P (2001). Rapid Signaling at Inhibitory Synapses in a Dentate Gyrus Interneuron Network.. J Neurosci.

[8] Wang X-J, Buzsáki G (1996). Gamma Oscillation by Synaptic Inhibition in a Hippocampal Interneuronal Network Model.. J Neurosci.

[9] Traub RD, Pais I, Bibbig A, LeBeau FEN, Buhl EH (2003). Contrasting roles of axonal (pyramidal cell) and dendritic (interneuron) electrical coupling in the generation of neuronal network oscillations.. Proc Natl Acad Sci U S A.

[10] De Zeeuw CI, Chorev E, Devor A, Manor Y, Van Der Giessen RS (2003). Deformation of Network Connectivity in the Inferior Olive of Connexin 36-Deficient Mice Is Compensated by Morphological and Electrophysiological Changes at the Single Neuron Level.. J Neurosci.

[11] Choi S, Yu E, Kim D, Urbano FJ, Makarenko V (2010). Subthreshold membrane potential oscillations in inferior olive neurons are dynamically regulated by P/Q- and T-type calcium channels: a study in mutant mice.. J Physiol (London).

[12] Leznik E, Llinás R (2005). Role of Gap Junctions in Synchronized Neuronal Oscillations in the Inferior Olive.. J Neurophysiol.

[13] Long MA, Deans MR, Paul DL, Connors BW (2002). Rhythmicity without Synchrony in the Electrically Uncoupled Inferior Olive.. J Neurosci.

[14] Chorev E, Yarom Y, Lampl I (2007). Rhythmic Episodes of Subthreshold Membrane Potential Oscillations in the Rat Inferior Olive Nuclei In Vivo.. J Neurosci.

[15] Khosrovani S, Van Der Giessen RS, De Zeeuw CI, De Jeu MTG (2007). In vivo mouse inferior olive neurons exhibit heterogeneous subthreshold oscillations and spiking patterns.. Proc Natl Acad Sci U S A.

[16] Llinás R, Sasaki K (1989). The Functional Organization of the Olivo-Cerebellar System as Examined by Multiple Purkinje Cell Recordings.. Eur J Neurosci.

[17] De Zeeuw C (1998). Microcircuitry and function of the inferior olive.. Trends Neurosci.

[18] Jacobson GA, Rokni D, Yarom Y (2008). A model of the olivo-cerebellar system as a temporal pattern generator.. Trends Neurosci.

[19] Swain RA, Kerr AL, Thompson RF (2011). The cerebellum: a neural system for the study of reinforcement learning.. Front Behav Neurosci.

[20] Ito M (2005). Bases and implications of learning in the cerebellum — adaptive control and internal model mechanism.. Prog Brain Res.

[21] Raymond JL, Lisberger SG, Mauk MD (1996). The Cerebellum: A Neuronal Learning Machine?. Science.

[22] Devor A, Yarom Y (2002). Generation and Propagation of Subthreshold Waves in a Network of Inferior Olivary Neurons.. J Neurophysiol.

[23] Chorev E, Manor Y, Yarom Y (2006). Density is destiny–on [corrected] the relation between quantity of T-type Ca2+ channels and neuronal electrical behavior.. CNS Neurol Disord Drug Targets.

[24] Jacobson GA, Lev I, Yarom Y, Cohen D (2009). Invariant phase structure of olivo-cerebellar oscillations and its putative role in temporal pattern generation.. Proc Natl Acad Sci U S A.

[25] Kistler WM, De Zeeuw CI (2005). Gap junctions synchronize synaptic input rather than spike output of olivary neurons.. Prog Brain Res.

[26] Sherman A, Rinzel J (1992). Rhythmogenic effects of weak electrotonic coupling in neuronal models.. Proc Natl Acad Sci U S A.

[27] Leznik E, Makarenko V, Llinás R (2002). Electrotonically Mediated Oscillatory Patterns in Neuronal Ensembles: An In Vitro Voltage-Dependent Dye-Imaging Study in the Inferior Olive.. J Neurosci.

[28] Loewenstein Y, Yarom Y, Sompolinsky H (2001). The generation of oscillations in networks of electrically coupled cells.. Proc Natl Acad Sci U S A.

[29] Schweighofer N, Doya K, Kawato M (1999). Electrophysiological Properties of Inferior Olive Neurons: A Compartmental Model.. J Neurophysiol.

[30] Manor Y, Rinzel J, Segev I, Yarom Y (1997). Low-Amplitude Oscillations in the Inferior Olive: A Model Based on Electrical Coupling of Neurons With Heterogeneous Channel Densities.. J Neurophysiol.

[31] Sotelo C, Llinas R, Baker R (1974). Structural study of inferior olivary nucleus of the cat: morphological correlates of electrotonic coupling.. J Neurophsyiol.

[32] Devor A, Yarom Y (2000). GABAergic modulation of olivary oscillations.. Prog Brain Res.

[33] Manor Y, Yarom Y, Chorev E, Devor A (2000). To beat or not to beat: A decision taken at the network level.. J Physiol (Paris).

[34] Schweighofer N, Doya K, Fukai H, Chiron JV, Furukawa T (2004). Chaos May Enhance Information Transmission in the Inferior Olive.. Proc Natl Acad Sci U S A.

[35] Bal T, McCormick DA (1997). Synchronized Oscillations in the Inferior Olive Are Controlled by the Hyperpolarization-Activated Cation Current Ih.. J Neurophysiol.

[36] De Zeeuw CI, Lang EJ, Sugihara I, Ruigrok TJH, Eisenman LM (1996). Morphological Correlates of Bilateral Synchrony in the Rat Cerebellar Cortex.. J Neurosci.

[37] Placantonakis DG, Bukovsky AA, Aicher SA, Kiem H-P, Welsh JP (2006). Continuous Electrical Oscillations Emerge from a Coupled Network: A Study of the Inferior Olive using Lentiviral Knockdown of Connexin36.. J Neurosci.

[38] Lang EJ (2002). GABAergic and Glutamatergic Modulation of Spontaneous and Motor-Cortex-Evoked Complex Spike Activity.. J Neurophysiol.

[39] Llinas R, Baker R, Sotelo C (1974). Electrotonic coupling between neurons in cat inferior olive.. J Neurophysiol.

[40] Uusisaari M, De Schutter E (2011). The Mysterious Microcircuitry of the Cerebellar Nuclei.. J Physiol (London).

[41] Zeeuw CID, Hoebeek FE, Bosman LWJ, Schonewille M, Witter L (2011). Spatiotemporal firing patterns in the cerebellum.. Nat Rev Neurosci.

[42] Vervaeke K, Lőrincz A, Gleeson P, Farinella M, Nusser Z (2010). Rapid Desynchronization of an Electrically Coupled Interneuron Network with Sparse Excitatory Synaptic Input.. Neuron.

[43] Dugué GP, Brunel N, Hakim V, Schwartz E, Chat M (2009). Electrical Coupling Mediates Tunable Low-Frequency Oscillations and Resonance in the Cerebellar Golgi Cell Network.. Neuron.

[44] Pfeuty B, Mato G, Golomb D, Hansel D (2003). Electrical Synapses and Synchrony: The Role of Intrinsic Currents.. J Neurosci.

[45] Solinas S, Forti L, Cesana E, Mapelli J, Schutter ED (2007). Computational reconstruction of pacemaking and intrinsic electroresponsiveness in cerebellar golgi cells.. Front Cell Neurosci.

[46] Smeal RM, Ermentrout GB, White JA (2010). Phase-response curves and synchronized neural networks.. Philos Trans R Soc Lond B Biol Sci.

[47] Izhikevich E (2007). Dynamical Systems in Neuroscience: The Geometry of Excitability and Bursting..

[48] Hines ML, Davison AP, Muller E (2009). NEURON and Python.. Front Neuroinform.

