# Supplementary material for: The Generation of Phase Differences and Frequency Changes in a Network Model of Inferior Olive Subthreshold Oscillations
Source: PLoS Comput Biol. 2012 Jul 5;8(7):e1002580. doi: 10.1371/journal.pcbi.1002580 (PMC3390386; doi:10.1371/journal.pcbi.1002580)
Supplement: Text S1 — Additional information about the robustness of the model, modulation of network frequencies and the range of frequencies at which (a pair of) IO model neurons can oscillate. (DOC) [file pcbi.1002580.s001.doc]

Supporting Text S1

Robustness

We presented a biophysical mechanism for the generation of subthreshold oscillations, modulation of oscillatory frequencies and the emergence of stable phase differences between individual neurons. To address the robustness of this mechanism we had to show that the network dynamics that emerge in the "4 clusters x 12 neuron" network is not anecdotal. Therefore we establish a working definition of robustness, and show that networks instantiated with various values for the free parameters (e.g., number of clusters and cluster size) exhibit dynamics that are in line with our definition of robustness.

Recall from the main text that we defined synchronized oscillations as oscillations that have the same time scale. Thus, we call a network synchronized when all the neurons inside the network oscillate at the same frequency. However, a (by this definition necessarily stable) phase difference can occur. We then say that the found dynamics are robust when we can also find networks with various numbers of cluster and of cluster sizes that satisfy our constraints and give rise to synchronized oscillations in the whole network.

We then demonstrated that the mechanism is also robust to larger ranges of clusters and cluster sizes. We ran simulation with networks of random number of clusters (8 to 20 clusters) and random network sizes (8 to 20 neurons). In Figure S1A, the numbers of neurons per cluster is identical in the whole network (For instance, a "11 cluster x 17 neurons"). In Figure S1B, the number of neurons per cluster is (potentially) different for each cluster (For instance, a network with 8 clusters can potentially have 8 distinct numbers of neurons per cluster). The dynamics in these networks follow the dynamics as explained in the main text. Hence, the network is robust to the number of clusters and neurons per cluster, and also to distinct cluster sizes in the network.

Frequency modulation in a pair of IO model neurons

Theoretical treatment

The theoretical treatment is borrowed from [1]. Consider the case where two neurons containing g_l and g_{Ca} are connected by weak electrical coupling, and, the value V_1 is mainly determined by g_l because g_l is much larger than g_{gap} (= 1/ R_{c1}) and g_{Ca}. Then, V_1 is dictated by g_l and quickly relaxes to E_l. The equation describing the V_2 can then be rewritten, without V1 because $V_1 \approx E_l$, as:

$$\frac{dV_2}{dt} = -\frac{1}{Cm}\left[I_{Ca} + g_l(V_2 - E_l) - g_{gap}(E_l - V_2)\right]$$

$$\frac{dV_2}{dt} = -\frac{1}{Cm}\left[I_{Ca} + g_l(V_2 - E_l) + g_{gap}(V_2 - E_l)\right]$$

$$\frac{dV_2}{dt} = -\frac{1}{Cm}\left[I_{Ca} + (g_l + g_{gap})(V_2 - E_l)\right]$$

Hence, in the case V_1 is dictated by the leak reversal E_l, coupling neuron #1 and #2

will have the same effect as adding more leak conductance (proportional to g_{gap}) to neuron #2. Because adding g_l to a neuron may change the frequency of the neuron, coupling two neurons under the stated condition can change the frequency. More specifically, in this particular case, the exact frequency change is solely dependent on strength of g_{gap} which moves the neuron horizontally in the g_l-g_{Ca} plane of Figure 1A and S2B. A similar argument can be constructed for the case in which V1 is mainly determined by the density of g_{Ca}.

Empirical treatment

We also empirically investigated the effect of the coupling strength on the frequency of the synchronized oscillation in a pair of neurons. To this end we uniformly sampled a large number of model-neuron pairs from experimentally observed ranges for the conductances and selected pairs in which both neurons spontaneously oscillated. Then, we coupled these pairs with a wide variety of coupling conductances (while maintaining CC < 20%) and recorded the coupling coefficients and frequencies at which both neurons oscillated in synchrony. Figure S3A illustrates the diversity of frequencies (in Hz) at which the neurons can oscillate in synchrony as a function of the difference between both uncoupled neurons (in Hz). For example, 3 on the x-axis means that the uncoupled neurons were 3 Hz apart (e.g., 6 and 9 Hz, or 4 and 7 Hz, etc…), while 3 on the y-axis means that both coupled neurons could oscillate over a range of 3 Hz depending on the exact coupling conductances (e.g., between 5 and 8Hz, or between 8 and 11 Hz, etc…). It can be seen that with increasing difference between the pairs, the pairs can display synchronized oscillations at a broader range of frequencies. However, if the difference becomes very large (> 5.5 Hz) the range of possible frequencies does not follow this pattern anymore because two neurons that are far apart in their conductance densities can either (i) synchronize at a limited number of frequencies only (hence a small range), or, (ii) synchronize at the frequency of either neuron (thus on a range which is similar to the difference in frequency between the neurons). Figure S3B illustrates the relative number of pairs not reaching synchrony for any of the tested coupling conductances as a function of the difference between the frequencies of the individual neurons. Clearly, the larger the difference between both neurons, the harder it becomes to synchronize under the weak coupling constraints. In Figures S3C-E we show three representative neuron pairs and the resulting frequencies at which synchronized oscillations occur as a function of the coupling strength. The red and blue y-axes represent the coupling coefficient measured in either direction while the x-axis denotes the frequency. Each dot represents a particular tested coupling conductance at which the pair oscillated in synchrony. The black boxes indicate the frequency of both neurons without coupling. Due to i) differential input resistances and ii) asymmetric coupling conductances CC1 and CC2 need not to be the same. In Figure S3C both original neurons are far apart and reach synchrony only at limited coupling strength. The pair from Figure S3D can span almost the entire range of frequencies between both neurons depending on the exact coupling strength. Finally, in Figure S3E, only half of frequency spectrum between both neurons can be reached and synchrony is only obtained at limited coupling strengths. We thus identified a robust mechanism stating that a coupled pair of "similar" neurons can oscillate synchronously under a wide range of coupling strengths (not exceeding their difference), and, that dissimilar pairs synchronize more difficult if at all. (Note that "similar" is used here in a much broader sense than in the main manuscript)

Propagating waves of activity

From a study with an *in-vitro* IO preparation, spontaneously propagating waves of activity were reported [2]. It was noted that under unperturbed conditions a phase-difference was measured between neurons in the same slice while they oscillated at the same frequency. Here we argue that these waves result from spreading of activity in the IO network due to phase differences between clusters. In our network model we also have sustained phase-differences, which can be attributed to clustering of the network. For visualization purposes we also generated a pseudo-random network consisting of 20 clusters with 20 neurons. Then, by arranging the cluster according to their resting membrane potential (viz., in order of increasing/decreasing Ca^{2+}) and normalizing the membrane potential of each neuron to be on the range [-1,1] we can visualize a clear propagation of activity in our network. It can be seen that the activity spreads quickly inside the cluster due to the dense connectivity and travels to other clusters through sparse connections. Then again, the activity spreads inside the connected cluster and the same process ensures that activity spreads from cluster to cluster. The supplemental video shows this process in detail. Figure S4 is taken from the “20 clusters x 20 neurons” network and illustrates the phase-map (S4A) and the cross-correlation between the clusters (S4B); indicating that the phase-differences are stable over time.

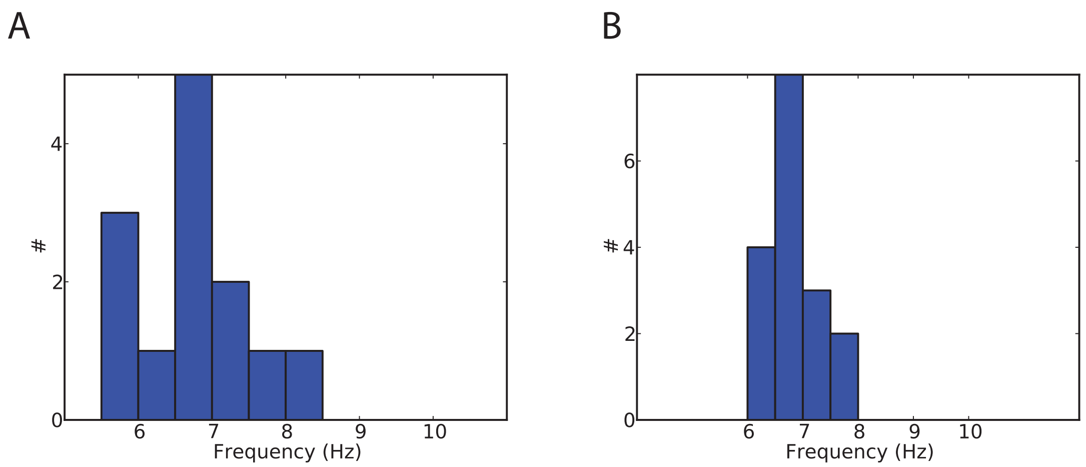

Figure S1: Robustness of the network dynamics as seen in the range of frequencies. A: Pseudo-random network with a random number of clusters (8 to 20 clusters) and neurons per clusters (8 to 20 neurons). In A, all clusters have the same number of neurons in one particular network. B: As in A but all clusters contain a random number of neurons. The range of frequencies at which the network exhibits coherent oscillations is the same and spans a range of 3 Hz.

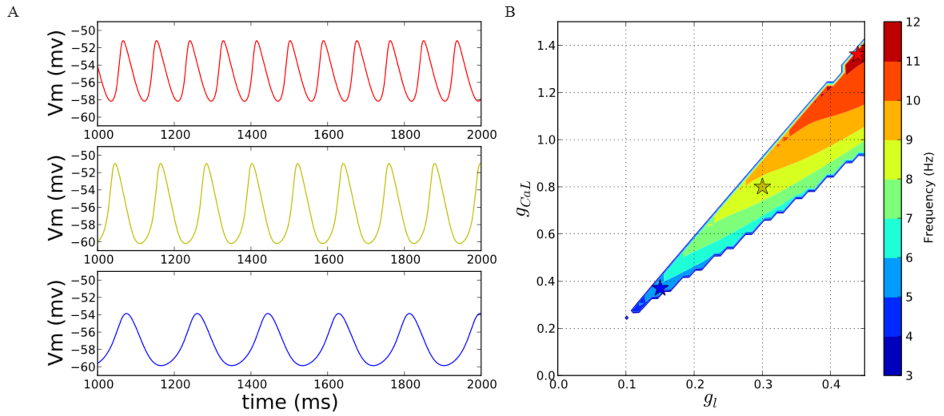

Figure S2: Single IO model neurons can oscillate at different frequencies. A: illustrations of different oscillations resulting from the same model neuron with differential densities of g_l and g_{Ca}. The color of the trace in A corresponds to the location in B, in which the exact quantities of g_l and g_{Ca} are indicated by a star. B: g_l-g_{Ca} map indicating the frequency of the spontaneous oscillations as a function of the g_l and g_{Ca} conductances.

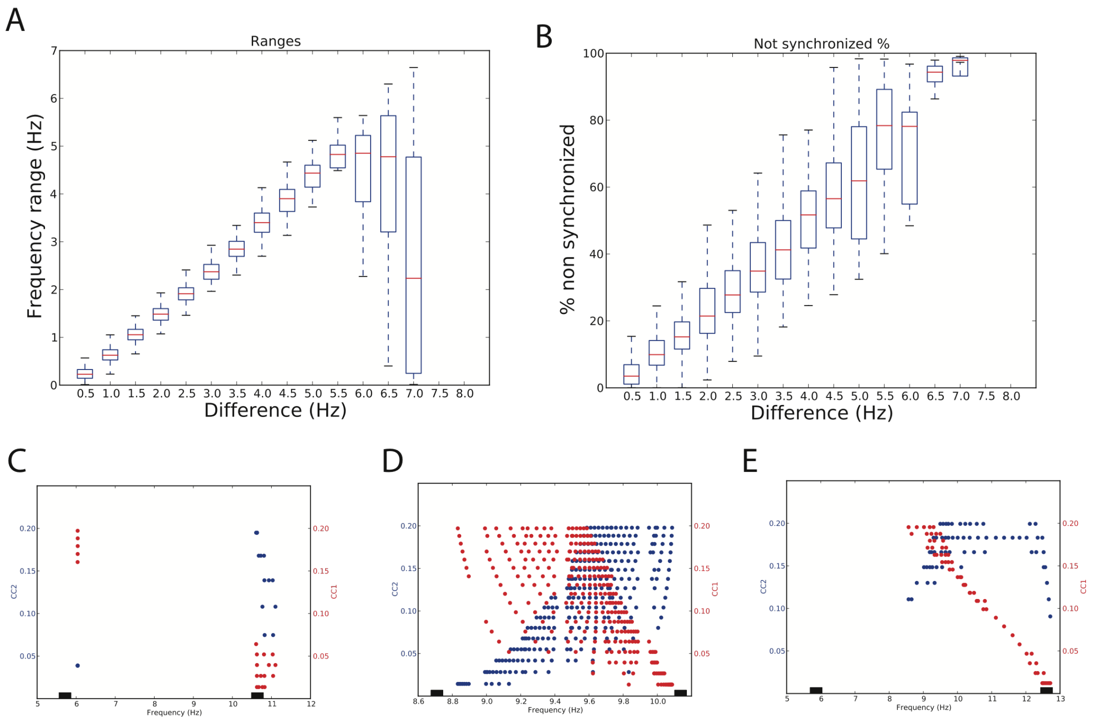

Figure S3: Coupling strength sets the frequency of the synchronized oscillation in a pair of neurons. See main Supplementary text.

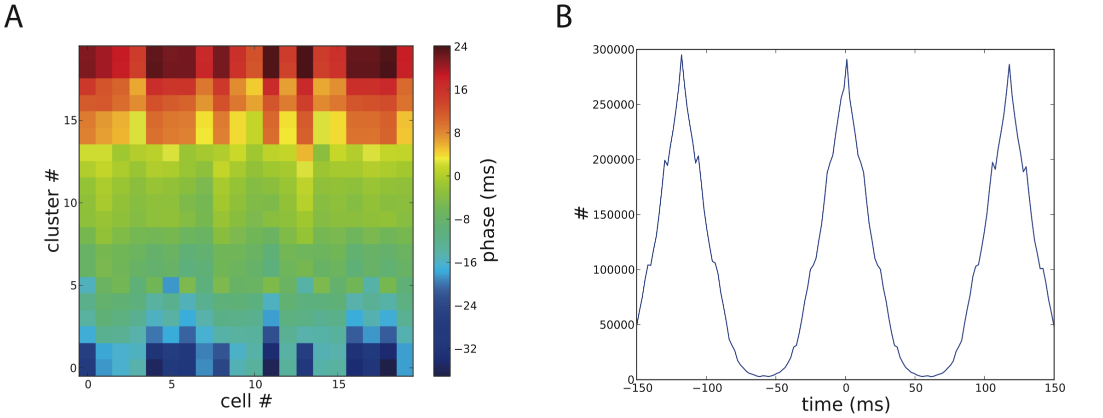

Figure S4: Presented IO network model dynamics are robust to the number of neurons per cluster and the number of cluster. Illustrated summarized data from a "20 clusters x 20 neurons" network. A: Phase-map color-coding the phase differences (here expressed in absolute time) between a reference neuron in the network (having 0 ms) and all other neurons in the network. B: cross-correlation between all neurons of the network. The main oscillatory frequency can be seen and the width of the peaks represents phase differences; the peaks are the same and hence the phase difference is stable over time. Supplemental video S1 shows the dynamics in this network and clearly shows the propagating wave of activity.

References

1. Manor Y, Rinzel J, Segev I, Yarom Y (1997) Low-Amplitude Oscillations in the Inferior Olive: A Model Based on Electrical Coupling of Neurons With Heterogeneous Channel Densities. J Neurol Neurophysiol 77: 2736–2752.
2. Devor A, Yarom Y (2002) Generation and Propagation of Subthreshold Waves in a Network of Inferior Olivary Neurons. J Neurol Neurophysiol 87: 3059–3069.
